# Evaluation of diagnostic criteria for IgG4-related tubulointerstitial nephritis

**DOI:** 10.1186/s13000-015-0311-3

**Published:** 2015-07-01

**Authors:** Xuanli Tang, Bin Zhu, Riping Chen, Yunqin Hu, Yinghua Zhang, Xiaoling Zhu, Hongyu Chen, Yongjun Wang

**Affiliations:** Department of Nephrology (Key laboratory of Zhejiang province, management of kidney disease), Hangzhou hospital of traditional Chinese medicine, Hangzhou, 310007 China; Department of Pathology, Zhejiang Academy of Medical Science, Hangzhou, 310007 China

**Keywords:** Diagnostic criteria, IgG4-related tubulointerstitial nephritis, IgG4, Pathological features, Immunohistochemistry

## Abstract

**Background:**

IgG4-TIN is the most common pattern of renal involvement in IgG4-related disease. There are several proposed diagnostic criteria of IgG4-TIN recently. Two of them proposed by the Mayo Clinic and JSN are predominant. However, histopathological criteria of the number of IgG4+ plasma cells and several histological features are still under discussion due to low amount of tissue in renal biopsy specimens and low frequency of this kind of specimens. We aimed to screen IgG4-TIN on archived renal biopsy samples and evaluated the application of two proposed diagnostic criteria.

**Methods:**

We selected 480 interstitial inflammation samples for light and electron microscopy and immunohistochemistry of CD138, IgG and IgG4 test. The Mayo Clinic proposed criteria diagnosed high-probability IgG4-TIN and JSN criteria confirmed IgG4-TIN.

**Results:**

Twelve high-probability IgG4-TIN were screened by histology, imaging, serology and other organ involvement according to the Mayo Clinic proposed criteria. The previous principal pathological diagnoses were IgAN (n=4), CreGN (n=4), tubulointerstitial nephritis (n=3) and LN (n=1). Three cases showed storiform fibrosis and a bird’s eye pattern. The distribution of IgG4+ plasma cells was focal, multifocal or diffuse, with a mixed mild, moderate or strong stainingpattern. Their treatment and clinical outcomes varied depending on different levels of proteinuria, serum creatinine, eGFR and original glomerular disease presentation. Therefore, we applied strict histological criteria of storiform fibrosis and evenly distributed IgG4+ plasma cells by JSN to confirm typical IgG4-TIN. Two cases were finally diagnosed as real IgG4-TIN. One was previously diagnosed as idiopathic interstitial nephritis with rapid response to corticosteroid therapy. The other was CreGN with immune complex deposits, which had poor outcome and long-term hemodialysis.

**Conclusions:**

IgG4-TIN might present concurrently with glomerular disease. The proposed criteria by the Mayo Clinic is flexible, sensitive, and superior in the identification of early-stage or atypical IgG4-TIN, with enhanced risk of misdiagnosis as compared to the proposed criteria by JSN, which is stricter, more specific, and might overlook early-stage or atypical IgG4-TIN. We propose a new set of criteria to improve pathologist-derived diagnosis.

## Introduction

IgG4-RD is recognized as a systemic autoimmune disease that is characterized by significant lymphoplasmacytic infiltration of IgG4 positive plasma cells, with obliterative phlebitis and storiform fibrosis leading to organ swelling or nodular lesions [1–3]. It was first recognized as sclerosing or AIP, which usually occurs with multi-organ involvement. Single organ injury such as kidney damage was reported occasionally [2–6]. IgG4-TIN was the most common pattern of renal involvement [7–11]. There were several proposed diagnostic criteria of IgG4-TIN in recent years, most of which emphasized histological features and rich IgG4+ plasma cells as indispensable criteria [3, 11, 12]. Nevertheless, the suitable cut-off values of IgG4+ plasma cells and the diagnostic strength of other specific histopathologic features are still debated in literature, also due to low amount of tissue in renal biopsy specimens and low frequency of this kind of specimens. In addition, IgG4 analysis in renal biopsy was not routinely performed previously, thus IgG4-TIN awareness was suffered and it would be easily misdiagnosed, especially when the morphological appearance was atypical. In this study, we retrospectively screened IgG4-TIN from archived renal biopsy samples, analyzed their clinical pathological characteristics and evaluated the utility of two proposed diagnostic criteria to identify their potential advantages and disadvantages.

## Materials and Methods

### Patients’ selection

Patients with sufficient acute or chronic interstitial inflammation (the frequency of inflammatory cells that were > 25 % within the cortical interstitium) by light microscopy were enrolled in this study from April 2008 through December 2013, irrespective of the presence or absence of glomerular disease. Altogether, 480 patients who were first admitted as renal injury without any remarkable medical history were studied following approval by the ethical committees of Hangzhou Hospital of Traditional Chinese Medicine.

HE-stained slides were reviewed by two pathologists. Cases were selected by lightmicroscopy as having an average plasma cell count of more than 5 plasma cells in at least 3 HPF fields [12]. Sections from the corresponding paraffin-embedded tissue blocks were recut and immunostained for the following antibodies: CD138 (#IR642; 1:50; Dako Cytomation, Glostrup, Denmark), IgG (#A0423; 1:250; Dako Cytomation, Glostrup, Denmark) and IgG4 (#AU009; 1:500; Binding Site, Birmingham, UK). Antigen was retrieved by EDTA solution as well as gastric enzyme, and Elivision system was applied in IHC detection. The positive cell counting was calculated as an average number per HPF over three fields. Abundant plasma cells were defined as more than 20/HPF. Suspected IgG4-TIN were defined as IgG4 positive cells > 10/HPF or IgG4/IgG positive cells >40 %.

### Screening for high-probability IgG4-TIN

High-probability IgG4-TIN cases were screened according to the criteria proposed by the Mayo Clinic as shown in Table 1 [12]. Clinical and laboratory features including age, gender, microscopic hematuria (−, <3; ±, 3–10; + 11–20; 2+, 21–40; 3+, 41–100; 4+, >100), 24-hour proteinuria, albumin, SCr, eGFR and IgG levels were then analyzed. The presence of eGFR was calculated by the mordification of diet in renal disease (MDRD). Histological features, determined on the basis of previous pathological studies of IgG4-RD were reanalyzed [3, 9, 13–17]. Immune complex deposits in TBM were observed by IgG test and electron microscopy.

**Table 1:** Two proposed criteria for IgG4-TIN by the Mayo Clinic and JSN

Criterion	The Mayo Clinic criteria	JSN criteria
Histology	Plasma cell-rich TIN with >10 IgG4+ plasma cells/HPF field in the most concentrated field (mandatory criterion)	a. Dense lymphoplasmacytic infiltrate with >10 IgG4+ plasma cells/HPF and/or IgG4/IgG+ plasma cell ratio of >40 %
	TBM immune complex deposits by immunofluorescence, immunohistochemistry, and/or electron microscopy	b. Characteristic storiform fibrosis
Imaging	Small peripheral low-attenuation cortical nodules, round or wedge-shapped lesions, or diffuse patchy involvement	Multiple low-density lesions on enhanced CT, diffuse kidney enlargement, hypovascular solitary nodule, hypertrophic lesion of the renal pelvic wall
Serology	Elevated serum IgG4 or total IgG level	Elevated serum IgG4 or total IgG level
Clinical features	None	Clinical or laboratory evidence of kidney damage
Other organ involvement	Characteristic findings of IgG4-RD in other organs	Characteristic findings of IgG4-RD in other organs
Definite IgG4-TIN	The histologic feature and at least one other feature from imaging, serology or other organ involvement	The histologic feature (a and b) and at least two of other features from imaging, serology or other organ involvement

### Confirmation of IgG4-TIN

The histological criterion proposed by JSN was much stricter as shown in Table 1 [18]. It emphasized that storiform fibrosis was an essential criterion, and that IgG4 positive cells should be marked and densely stained [18, 19]. We followed this guideline to confirm IgG4-TIN since lesions were numerous in our study, including many different glomerular diseases that might lead to a misdiagnosis of the underlying condition. Therefore confirmed IgG4-TIN was screened from a high-probability IgG4-TIN group according to the criteria proposed by JSN.

### Statistical analysis

Statistical analysis was performed with SPSS software version 17.0. Normally distributed data were expressed as mean ± SD. The clinical characteristics between two groups were compared by Student’s t test. Categorical variables of histological features between two groups were expressed as percentages and were compared by Chi-square test or Fisher’s exact test. Correlations were assessed by Spearman’s rank correlation to estimate the relationship of the clinical characteristics associated with IgG4 positive cells and the stage of interstitial fibrosis. P-values less than 0.05 were considered statistically significant.

## Results

### Cases of suspected IgG4-TIN

**Fig. 1:**
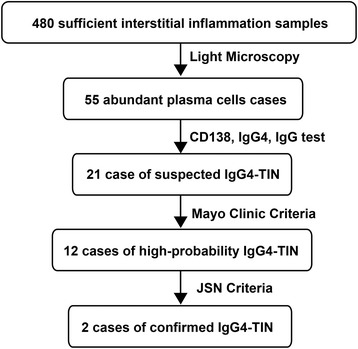
The procedure of screening cases

The procedure of screening cases is shown in Fig. 1. Herein, 480 samples with sufficient interstitial inflammation that accounted for 24.2 % of total renal biopsy specimens were selected in our study. After re-observing these specimens by light microscopy, 55 cases demonstrating abundant plasma cells were screened. Furthermore, 21 cases of suspected IgG4-TIN were screened from the previous group by CD138, IgG and IgG4 test, which accounted for 38.2 % of abundant plasma cell cases and 4.4 % of sufficient inflammation cases. The average number of CD138, IgG and IgG4 positive cells were 75.8 ± 34.9, 34.0 ± 21.3 and 19.9 ± 13.3 respectively. Their predominant pathological diagnoses were IgAN, CreGN and TIN respectively, whilst others were FSGS, LN, MN and SS.

### Cases of high-probability IgG4-TIN

According to the proposed criteria of the Mayo Clinic [12], twelve cases were screened at least for one other feature from the imaging, serology, or other organ involvement, which accounted for 57.1 % of suspected cases of IgG4-TIN. The average age was 52 years (range, 30 to 76 years). Six cases were male and the other six were female. Patients were much older (p < 0.01) whereas the incidence of proteinuria was lower (p < 0.05) than that of excluded cases (please see Table 2). In addition, Table 3 showed clinical and pathological features of twelve cases. Ten cases (or 83.3 %) had increased serum IgG levels; three cases (or 25 %) showed diffuse enlargement of both kidneys, six cases (or 50 %) presented with pulmonary interstitial fibrosis and one case had lymph node enlargement. All but one of the cases manifested acute (n = 6) or chronic (n = 5) renal failure.

**Table 2:** The comparison of clinical characteristics between the high-probability IgG4-TIN group and excluded group in abundant plasma cells samples

	High-probability IgG4-TIN (n = 12)	Excluded (n = 43)	P value
Female gender (n (%))	6 (50 )	26 (60 )	0.152
Age (y)	52 (30–76)	37 (14–63)	0.003
Proteinuria (0.01-0.15 g/24 h)	1.04 (0.21 - 2.69)	3.12 (0.06 - 17.33)	0.043
Albumin (35–55 g/L)	32.8 (25.7 - 42.1)	31.8 (15–46)	0.682
Serum creatinine (44–132 μmol/L)	311.7 (46–953)	492.8 (105–1832)	0.070
eGFR (80-120 ml/min)	23.6 (5.4 - 65.3)	29.5 (3.2 - 143.1)	0.405

Data are expressed as mean (minimum, maximum), number (frequency)

**Table 3:** Clinical, radiographic, laboratory and pathological features of twelve high-probability IgG4-TIN cases

Pt No.	Age/Gender	Clinical Diagnosis	Proteinuria (0.01-0.15g/24h)	Hematuria	ALB (35-55 g/L)	SCr (44-132μmol/L)	eGFR (80-120ml/min)	Serum IgG (751-1560mg/dL)	Serum C3 (79-152 mg/dL)	Principal Pathological Diagnosis	IgG4 (cells/HPF)	IgG4/IgG (%)	Storiform fibrosis	Stage of Fibrosis	TBM Deposits	Imaging Findings	Other Organ Involvement
1	65 M	ARF	0.30	-	36	381	13.2	1910	67	Idiopathic TIN	14	23.3	-	0	-	Bilateral Enlarged	-
2	42 F	ARF, AAV	1.41	4+	35	447	9.9	2370	78	Pauci-immune CreGN	22	56.7	-	0	+	-	-
3	52 F	SLE, CRF	1.57	2+	30	273	15.5	3050	86	LN	11	84.6	-	1	+	-	PF
4	76 M	CGN, CRF	1.41	4+	33	380	11.9	1990	75	Immune complex CreGN	10	43.5	-	1	-	-	PF
5	62 M	CGN, CRF	1.17	-	30	234	25.6	4050	45	Idiopathic TIN	55	93.2	+	2	+	Bilateral Enlarged	PF Lymph node Enlarged
6	49 M	CGN, CRF	0.40	3+	37.6	138	52	1050	81	IgAN	12	32.2	-	1	+	-	PF
7	33 F	ARF	0.21	-	39.9	231	24.9	2010	123	Drug-induced TIN	22	58	-	1	+	-	-
8	59 M	ARF	0.60	3+	26.6	1108	10.7	1380	86	Anti-GBM CreGN	29	60.3	-	1	-	-	PF
9	36 F	CGN, CRF	2.69	3+	30.4	199	40.7	1910	121	IgAN	14	40.3	-	2	+	-	-
10	56 F	ARF, AAV	1.58	3+	26.9	1832	5.4	2120	80	Crescentic IgAN	20	95	+	1	+	-	PF
11	59 M	ARF	0.49	-	25.7	586	8.4	1860	106	Immune complex CreGN	52	91	+	2	+	Bilateral Enlarged	-
12	30 F	CGN,CKD2	0.61	-	42.1	105	65.3	1870	109	IgAN	25	63.7	-	2	+	-	-

CreGN, Crescentic Glomerulonephritis; AAV, antineutrophil cytoplasmic autoantibody-associated vasculitis; PF, pulmonary fibrosis; LN, lupus nephritis; IgAN, IgA nephropathy; GBM, glomerular basement membrane.

**Fig. 2:**
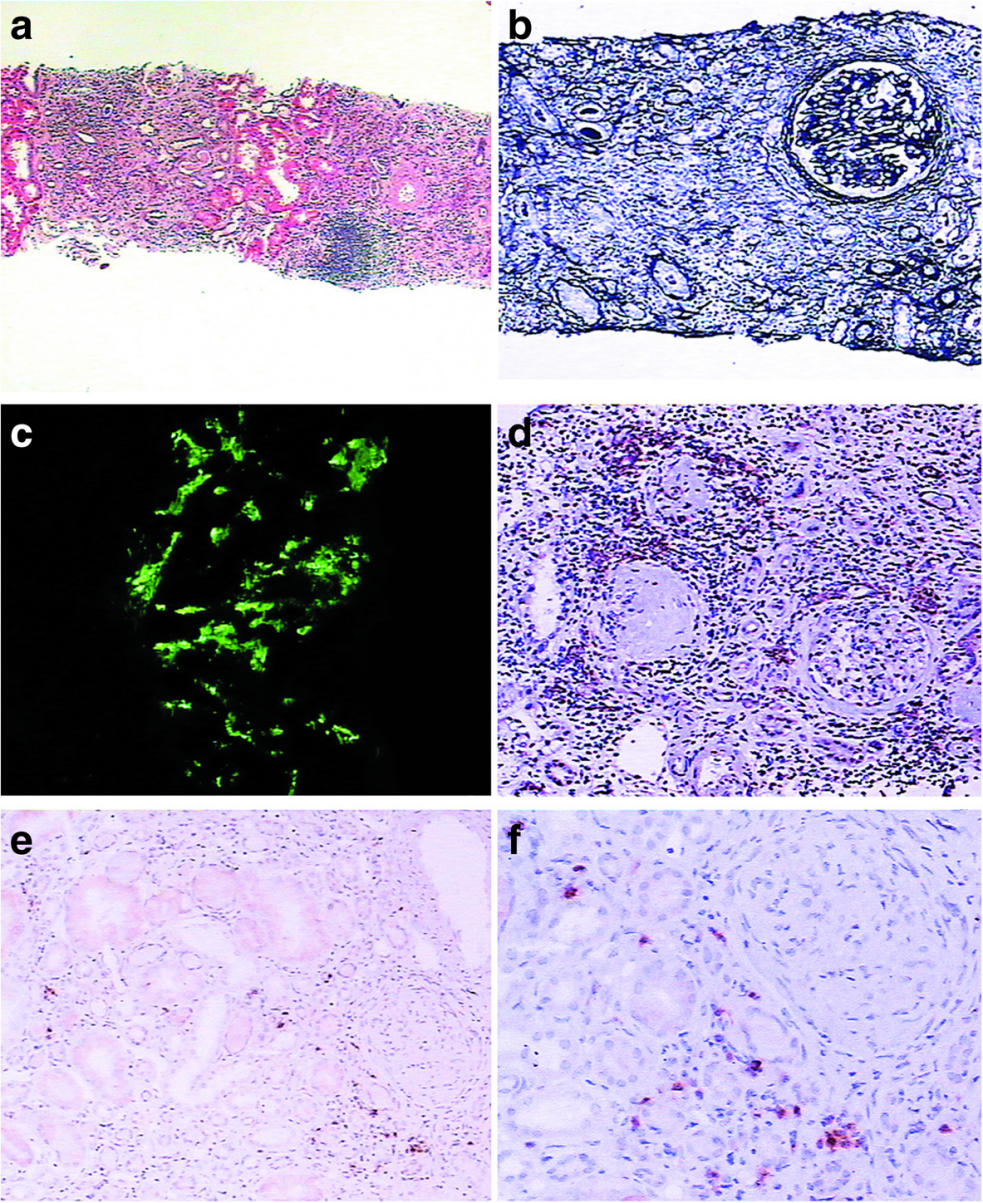
Histological features and immunohistochemistry findings of case twelve. **a**) Multi-focal fibrosis with obliteration of several tubules (×40) HE. **b**) Isolated glomeruli in the middle of fibrosis (×100) PAM. **c**) IgA deposits in mesengium (×200) IF. **d**) Multi-focally distributed plasma cells by CD138 test (×100) IHC. **e**) Focally scattered IgG4 positive cells by IgG4 test (×100) IHC. **f**) IgG4 test (×200) IHC

Originally, nine cases were diagnosed with glomerular diseases and three cases were diagnosed with TIN pathologically. Both CreGN and IgAN were predominant glomerular diseases among them. The others were idiopathic TIN, drug-induced TIN and LN (Table 3). Most of their histological features were non-specific; however, storiform fibrosis and a bird’s eye pattern were significantly more frequent than that in the excluded cases (p < 0.01; Table 4). Moreover, IgG4+ plasma cells were focal, multi-focal or evenly distributed, with mild, moderate or strongly stained mixed expression patterns (Fig. 2). Fibrosis was mild or moderate with multifocal distribution. The stage of interstitial fibrosis ranged from 0 to 2, and most cases were in stage 1. It was neither significantly correlated with the number of IgG4+ cells (r = 0.433, P = 0.160) nor TBM immune complex deposits (r = 0.426, P = 0.167). Both the number of IgG4 positive cells and the stage of interstitial fibrosis had no significant correlation with clinical laboratorial characteristics (age, hematuria, proteinuria, albumin, SCr and eGFR) (P > 0.05). Tubulitis presented with significant differences between both groups, though it was not included into specific features of IgG4-TIN (p < 0.05; Table 4). Moreover, IgG or electron dense deposits in TBM were more frequently observed in the high-probability cases than excluded cases (p < 0.05; Table 4).

**Table 4:** Pathological features of confirmed IgG4-TIN, high-probability IgG4-TIN and both excluded groups in abundant plasma cells samples

	Abundant plasma cells samples			High-probability IgG4-TIN		
Pathological features	High-probability IgG4-TIN (n = 12)	Excluded (n = 43)	P value 1	Confirmed IgG4-TIN (n = 2)	Unconfirmed IgG4-TIN (n = 10)	P Value 2
Inflammation infiltrating renal capsule	4 (33.3 )	11 (25.6)	0.594	1 (50)	3 (30)	0.584
Inflammation infiltrating renal medulla	6 (50)	23 (53.5)	0.831	1 (50)	5 (50)	1.000
Isolated fibrosis	6 (50)	3 (7)	0.000	2 (100)	4 (40)	0.121
Collagenous fibrosis	8 (66.7)	7 (16.3)	0.001	2 (100)	6 (60)	0.273
Storiform fibrosis	3 (25)	1 (2.3)	0.007	2 (100)	1 (10)	0.007
Lymphoid follicles	2 (16.7)	1 (2.3)	0.053	0 (0)	2 (20)	0.488
Granulomatous lesions	1 (8.3)	0 (0)	0.056	0 (0)	1 (10)	0.640
Necrotizing angiitis	1 (8.3)	1 (2.3)	0.326	0 (0)	1 (10)	0.640
Obliterative phlebitis	0 (0)	0 (0)	--	0 (0)	0 (0)	--
Eosinophil infiltration	8 (66.7)	18 (41.9)	0.128	2 (100)	6 (60)	0.273
Neutrophil infiltration	6 (50)	14 (32.6)	0.267	1 (50)	5 (50)	1.000
Tubulitis	9 (75)	16 (37.2)	0.020	2 (100)	7 (70)	0.371
Peritubular capillaritis	3 (25)	13 (30.2)	0.724	1 (50)	2 (20)	0.371
Tubular necrosis	8 (66.7)	18 (41.9)	0.128	0 (0)	8 (80)	0.091
TBM immune complex deposits	9 (75)	12 (27.9)	0.006	2 (100)	7 (70)	0.371

Data are expressed as number (frequency); P value 1: High-probability IgG4-TIN compared to Excluded; P value 2: Confirmed IgG4-TIN compared to unconfirmed; --: no statistics.

### Cases of confirmed IgG4-TIN

**Fig. 3:**
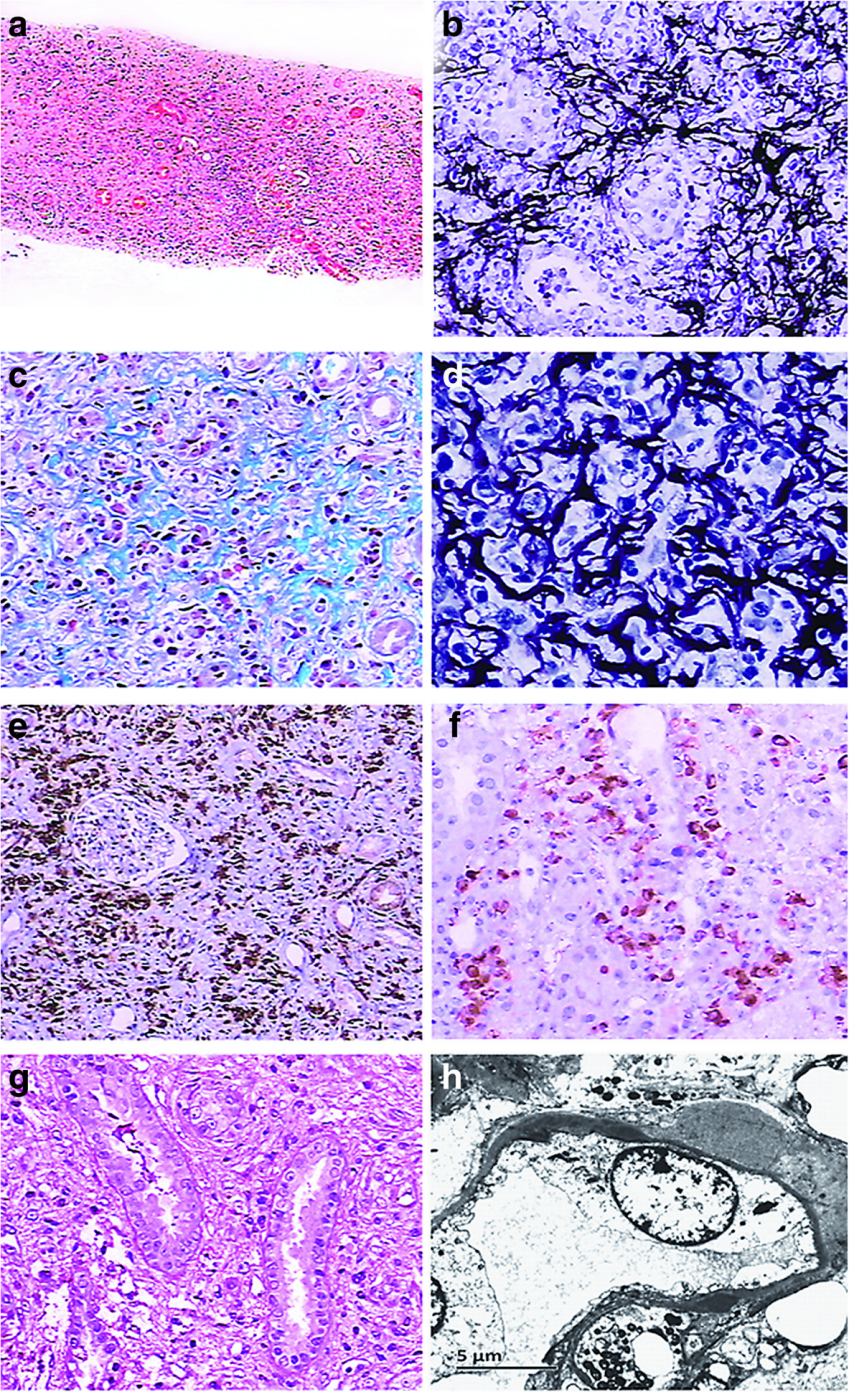
Pathological features of case five of histology, IHC and electron microscopy. **a**) Diffuse fibrosis with obliteration of most tubules and isolation of glomeruli (×40) HE. **b**) Typical storiform fibrosis with abundant inflammation cells (×200) PAM. **c**) Rich collagen fibrosis and characteristic bird’s-eye pattern (×200) Masson trichrome staining. **d**) Characteristic bird’s-eye pattern (×400) PAM. **e**) Abundant, evenly distributed, strong positive plasma cells in interstitium by CD138 test (×100) IHC. **f**) Abundant strong stained and evenly distributed IgG4+ plasma cells (>50 per HPF) in interstitium and tubular epithelial cells by IgG4 test (×200) IHC. **g**) Apparent TBM deposition of two tubules by IgG test (×200) IHC. **h**) Thickened TBM with scattered electron dense immune complex deposits (×4000) EM

In the high-probability IgG4-TIN group, all 12 cases presented with substantial histological differences, including the stage of fibrosis, the number of IgG4 positive cells and their distribution. Their treatment and clinical outcomes were absolutely different, and were dependent, at least in part, on proteinuria, serum creatinine, eGFR and presence of original glomerular disease. Therefore, we applied strict morphological criterion by JSN to confirm the diagnosis of IgG4-TIN and found three cases that met it. Two cases (case 5 and case 11) met all criteria of histology, imaging, serology and other organ involvement. The left case (case 10) met all criteria but the imaging criterion.. The histological feature of this case, such as storiform fibrosis, was mild and distributed focally. Moreover, IgG4 positive cells were unevenly distributed and moderately stained. Therefore, we checked through the finding of more references, in which it was recommended to identify the presence of dense, diffuse infiltrates of IgG4 positive cells >50/HPF, which were highly specific [20–24]. Moreover, an immunohistochemical study of IgG4-TIN from JSN showed the average number of IgG4 positive cells was 43.8/HPF [5]. Besides, Houghton DC and Troxell ML presented that the distribution of IgG4 positive cells were even and uniform in IgG4-TIN, while they were focal or multi-focal in various settings of interstitial nephritis [17]. Finally, we put the remained one case in the high-probability IgG4-TIN group, from which two cases were finally confirmed as IgG4-TIN. One of IgG4-TIN manifested ARF and the principal pathological diagnosis was immune complex CreGN. His serum ANCA was negative, which had no proof of AAV. The serum IgG4 level had decreased rapidly after a large dose of corticosteroid treatment, whereas his serum creatinine hardly decreased and hemodialysis was finally applied. Another case displayed chronic renal failure and the previous diagnosis was idiopathic TIN. Serum IgG4 level was undetectable at this time. Large dose of corticosteroids was prescribed and serum creatinine had decreased to normal level by one half month later. Both cases presented with a diffuse bird’s-eye pattern and focal storiform features [25] (Fig. 3), whereas there was only one case that showed specific fibrosis in ten left cases, which had significant difference (P < 0.01; Table 4). Moreover, IgG4+ plasma cells were presented at a frequency of more than 50 per HPF, which appeared as an evenly strong staining pattern with diffuse infiltration of the renal interstitium, while it was multifocal in left cases. No tubular necrosis was observed in confirmed cases, though it was frequently shown in the left cases.

## Discussion

The epidemiology and etiology of IgG4-TIN remain unclear. It has been hypothesized that autoimmune disorders and allergic reactions might be the heterogeneous causes [26–28]. However, this was opposed by several researchers. They demonstrated that IgG4 antibody inhibited activation of complement to suppress immune reactivity. Moreover, a specific allergen was never detected [29, 30]. Recently, it was reported that IgG4-TIN was associated with a hematological disorder [31]. Thus, the pathogenesis of this disease remains unclear.

The characteristic histological features such as storiform fibrosis and a bird’s-eye pattern were shown in more than 90 % of IgG4-TIN cases by JSN [18, 32]. By contrast, these presentations were shown in only 17.1 % of cases in the Mayo Clinic [12]. Therefore, several studies tried to define the stages of fibrosis to achieve consensus [19]. In our study, most of the high-probability IgG4-TIN cases showed that mild or moderate fibrosis and the typical storiform pattern was rarely observed. However, a bird’s-eye pattern was easier to find with numerous plasma cell nests encircled in fibrosis [25]. We presumed that this was an early characteristic feature of IgG4-TIN, which would progress to storiform fibrosis. In addition, immune complex deposition in TBM was reported to be more easily observed than specific fibrosis [12, 19]. In the present study, immune complex deposition was marginally more frequently observed than specific fibrosis in the high-probability IgG4-TIN group, although they all presented in the confirmed group. Some common features, such as extension of lesions into renal capsules and eosinophil infiltration strongly indicated IgG4-TIN; while tubulitis, peritubular capillaritis, necrotizing angiitis and neutrophil infiltration were negatively suggestive of IgG4-TIN [19, 25]. However, we found that only tubulitis was more frequent in high-probability IgG4-TIN than that in the excluded cases. Eosinophil infiltration was demonstrated to reproducibly be seen in IgG4-TIN [12, 13, 19], although it was not significantly different from other TIN cases such as allergic interstitial nephritis or acute CreGN in our study. This might be explained by that the lesions seen in our cases were much more variable, including many glomerular diseases with acute interstitial inflammation, thus both eosinophil and neutrophil infiltration were non-specific.

**Fig. 4:**
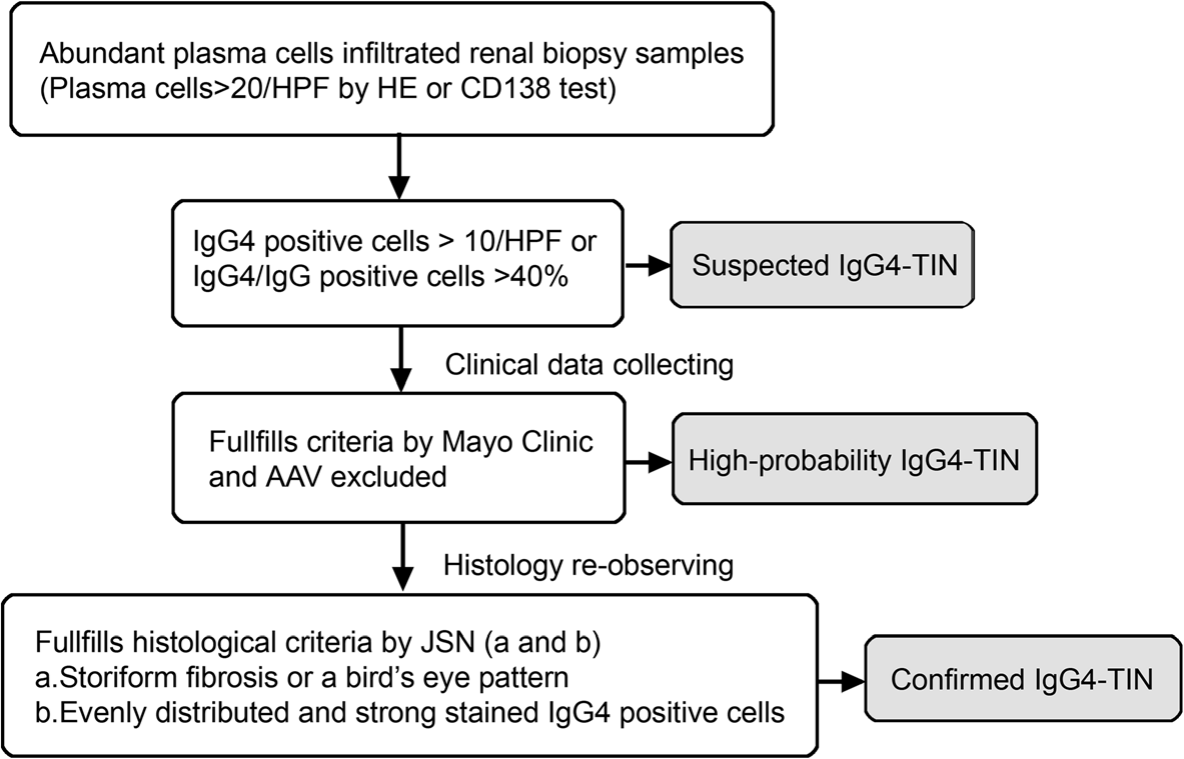
A new set of criteria of IgG4-TIN for pathologist

The clinical and pathological evidence of unconfirmed IgG4-TIN remain understudy. It’s unknown whether IgG4-TIN is concurrent with or earlier than glomerular lesions, or the second injury of glomerular diseases. In previous studies, some pauci-immune CreGN, DN, LN and MN met the criteria of IgG4 > 10 per HPF, but they were not real IgG4-TIN [12, 17]. The fibrosis and inflammatory lesions of these suspected cases were patchy or segregated, and IgG4+ plasma cells were focally and less densely distributed, which was different from the IgG4-TIN. In our study, cases were selected irrespective of glomerular disease, thus glomerular nephritis with interstitial IgG4 positive cells was included in high-probability IgG4-TIN, but their histological features had remarkable differences. Moreover, their reaction to treatment and prognosis varied considerably from each other. Six cases recovered well from poor renal function, two cases remained stable, and four cases progressed to end stage renal disease requiring long-term hemodialysis. Therefore we used the stricter criteria proposed by JSN and finally confirmed two cases. One had acute glomerular nephritis, which convinced us to believe that IgG4-TIN could co-exist with glomerulonephritis, wherein the prognosis was much worse and the patient depended on long-term hemodialysis. Although ten left high-probability cases met the proposed criteria by the Mayo Clinic, their clinical manifestations, histological features, treatment and prognosis varied from each other. Thus, we currently consider them to be unreal IgG4-TIN, although frequent follow-up will be necessary to verify. The proposed criteria by the Mayo Clinic are much looser and more sensitive, which makes it easier to determine early-stage or atypical IgG4-TIN, although it increases the risk of misdiagnosis. By contrast, the proposed criteria by JSN are much stricter and more specific, and yet early-stage or atypical IgG4-TIN might be easily missed. Therefore, we propose a new set of criteria for renal biopsy patients in Fig. 4, which would enable the pathologist to make more accurate diagnosis. Further, studies of more accumulated cases and long-term follow-up are necessary for etiology and proposing criteria of atypical IgG4-TIN.

## Conclusion

IgG4-TIN is easily missed without IgG4 test at the time of renal biopsy. The essential nature of increased IgG4 plasma cells is unclear and it will exist in various kidney diseases meanwhile. Strict histological criteria are helpful, and sometimes necessary in differentiation of IgG4-TIN.

## Abbreviations

IgG4-RD: IgG4-related disease
AIP: Autoimmune pancreatitis
IgG4-TIN: IgG4-related tubulointerstitial nephritis
HE: Hematoxylin and eosin
HPF: High power field
IHC: Immunohistochemistry
SCr: Serum creatinine
eGFR: Estimated glomerular filtration rate
TBM: Tubular basement membrane
JSN: The Japanese Society of Nephrology
IgAN: IgA nephropathy, CreGN, Crescentic glomerulonephritis
TIN: Tubulointerstitial nephritis
FSGS: Focal segmental glomerulous sclerosis
LN: lupus nephritis
MN: Membranous nephropathy
SS: Sjogren Syndrome
ARF: Acute renal failure
ANCA: Antineutrophil cytoplastic antibody
PAM: Periodic acid methenaming silver stain
IF: Immunofluorescence
AAV: ANCA-associated vasculitis

## References

[1] Kamisawa T, Okamoto A (2008). IgG4-related sclerosing disease. World J Gastroenterol.

[2] Masaki Y, Dong L, Kurose N, Kitagawa K, Morikawa Y, Yamamoto M (2009). Proposal for a new clinical entity, IgG4-positive multi-organ lymphoproliferative syndrome: analysis of 64 cases of IgG4-related disorders. Ann Rheum Dis.

[3] Deshpande V, Zen Y, Chan JK, Yi EE, Sato Y, Yoshino T (2012). Consensus statement on the pathology of IgG4-related disease. Mod Pathol.

[4] Hamano H, Kawa S, Horiuchi A, Unno H, Furuya N, Akamatsu T (2001). High serum IgG4 concentrations in patients with sclerosing pancreatitis. N Engl J Med.

[5] Takeda S, Haratake J, Kasai T, Takaeda C, Takazakura E (2004). IgG4-associated idiopathic tubulointerstitial nephritis complicating auto-immune pancreatitis. Nephrol Dial Transplant.

[6] Uchiyama-Tanaka Y, Mori Y, Kimura T, Sonomura K, Umemura S, Kishimoto N (2004). Acute tubulointerstitial nephritis associated with auto-immune-related pancreatitis. Am J Kidney Dis.

[7] Saeki T, Nishi S, Ito T, Yamazaki H, Miyamura S, Emura I (2007). Renal lesions in IgG4-related systemic disease. Intern Med.

[8] Tsubata Y, Akiyama F, Oya T, Ajiro J, Saeki T, Nishi S (2010). IgG4-related chronic tubulointerstitial nephritis without auto-immune pancreatitis and the time course of renal function. Intern Med.

[9] Saeki T, Nishi S, Imai N, Ito T, Yamazaki H, Kawano M (2010). Clinicopathological characteristics of patients with IgG4-related tubulointerstitial nephritis. Kidney Int.

[10] He TM, Qu LJ, Xie FL, Zheng ZY (2013). Pathologic features of IgG4 related diseases involving kidney and lymph node. J Diag Pathol.

[11] Zheng K, Li XM, Cai JF, Wen YB (2012). Analysis on urinary system lesions of IgG4-related disease. Chin J Nephrol.

[12] Raissian Y, Nasr SH, Larsen CP, Colvin RB, Smyrk TC, Takahashi N (2011). Diagnosis of IgG4-Related Tubulointerstitial Nephritis. J Am Soc Nephrol.

[13] Cornell LD (2012). IgG4-related kidney disease. Semin Diagn Pathol.

[14] Chari ST, Kloeppel G, Zhang L, Notohara K, Lerch MM, Shimosegawa T (2010). Histopathologic and clinical subtypes of autoimmune pancreatitis: the Honolulu consensus document. Pancreas.

[15] Cheuk W, Chan JK (2010). IgG4-related sclerosing disease: a critical appraisal of an evolving clinicopathologic entity. Adv Anat Pathol.

[16] Zen Y, Nakanuma Y (2010). IgG4-related disease. A cross-sectional study of 114 cases. Am J Surg Pathol.

[17] Houghton DC, Troxell ML (2011). An abundance of IgG4+ plasma cells is not specific for IgG4-related tubulointerstitial nephritis. Mod Pathol.

[18] Kawano M, Saeki T, Nakashima H, Nishi S, Yamaguchi Y, Hisano S (2011). Proposal for diagnostic criteria for IgG4-related kidney disease. Clin Exp Nephrol.

[19] Yoshita K, Kawano M, Mizushima I, Colvin RB, Smyrk TC, Takahashi N (2012). Light-microscopic characteristics of IgG4-related tubulointerstitial nephritis: distinction from non-IgG4-related tubulointerstitial nephritis. Nephrol Dial Transplant.

[20] Zhang L, Notohara K, Levy MJ, Chari ST, Smyrk TC (2007). IgG4-positive plasma cell infiltration in the diagnosis of autoimmune pancreatitis. Mod Pathol.

[21] Shrestha B, Sekiguchi H, Colby TV, Graziano P, Aubry MC, Smyrk TC (2009). Distinctive pulmonary histopathology with increased IgG4-positive plasma cells in patients with autoimmune pancreatitis: report of 6 and 12 cases with similar histopathology. Am J Surg Pathol.

[22] Kamisawa T, Funata N, Hayashi Y, Eishi Y, Koike M, Tsuruta K (2003). A new clinicopathological entity of IgG4-related autoimmune disease. J Gastroenterol.

[23] Dhall D, Suriawinata AA, Tang LH, Shia J, Klimstra DS (2010). Use of immunohistochemistry for IgG4 in the distinction of autoimmune pancreatitis from peritumoral pancreatitis. Hum Pathol.

[24] Kawano M, Mizushima I, Yamaguchi Y, Imai N, Nakashima H, Nishi S (2012). Immunohistochemical Characteristics of IgG4-Related Tubulointerstitial Nephritis: Detailed Analysis of 20 Japanese Cases. Int J Rheumatol.

[25] Yamaguchi Y, Kanetsuna Y, Honda K, Yamanaka N, Kawano M, Nagata M (2012). Characteristic tubulointerstitial nephritis in IgG4-related disease. Hum Pathol.

[26] Cornell LD (2010). IgG4-related tubulointerstitial nephritis. Kidney Int.

[27] Umehara H, Okazaki K, Masaki Y, Kawano M, Yamamoto M, Saeki T (2012). A novel clinical entity, IgG4-related disease (IgG4RD): general concept and details. Mod Rheumatol.

[28] Stone JH, Zen Y, Deshpande V (2012). IgG4-related disease. N Engl J Med.

[29] Van der Zee JS, van Swieten P, Aalberse RC (1986). Serologic aspects of IgG4 antibodies. II. IgG4 antibodies form small, nonprecipitating immune complexes due to functional monovalency. J Immunol.

[30] Nishi S, Imai N, Yoshida K, Ito Y, Saeki T (2011). Clinicopathological findings of immunoglobulin IgG4-related kidney disease. Clin Exp Nephrol.

[31] Malone AF, Sparks MA, Howell DN, Middleton JP, Smith SR, Lehrich RW (2013). IgG4-related tubulointerstitial nephritis associated with chronic lymphocytic leukemia. J Nephrol.

[32] Kawano M, Saeki T (2015). IgG4-related kidney disease–an update. Curr Opin Nephrol Hypertens.

